# Buzzfindr: Automating the detection of feeding buzzes in bat echolocation recordings

**DOI:** 10.1371/journal.pone.0306063

**Published:** 2024-08-20

**Authors:** Joël W. Jameson

**Affiliations:** WSP, Montreal, Quebec, Canada; Institute of Geographic Sciences and Natural Resources Research Chinese Academy of Sciences, CHINA

## Abstract

Quantification of bat communities and habitat heavily rely on non-invasive acoustic bat surveys the scope of which has greatly amplified with advances in remote monitoring technologies. Despite the unprecedented amount of acoustic data being collected, analysis of these data is often limited to simple species classification which provides little information on habitat function. Feeding buzzes, the rapid sequences of echolocation pulses emitted by bats during the terminal phase of prey capture, have historically been used to evaluate foraging habitat quality. Automated identification of feeding buzzes in recordings could benefit conservation by helping identify critical foraging habitat. I tested if detection of feeding buzzes in recordings could be automated with bat recordings from Ontario, Canada. Data were obtained using three different recording devices. The signal detection method involved sequentially scanning narrow frequency bands with the “Bioacoustics” R package signal detection algorithm, and extracting temporal and signal strength parameters from detections. Buzzes were best characterized by the standard deviation of the time between consecutive pulses, the average pulse duration, and the average pulse signal-to-noise ratio. Classification accuracy was highest with artificial neural networks and random forest algorithms. I compared each model’s receiver operating characteristic curves and random forest provided better control over the false-positive rate so it was retained as the final model. When tested on a new dataset, buzzfindr’s overall accuracy was 93.4% (95% CI: 91.5%– 94.9%). Overall accuracy was not affected by recording device type or species frequency group. Automated detection of feeding buzzes will facilitate their integration in the analytical workflow of acoustic bat studies to improve inferences on habitat use and quality.

## Introduction

Increased threats to bats including White Nose Syndrome, wind energy, and habitat loss have led to increased protections and conservation efforts for impacted species [1]. In light of recent advances in acoustic monitoring technology, these protections have generally consisted in increasing the scope of acoustic monitoring for bats [2–4]. Unfortunately, while the amount of data being collected is growing exponentially, the scope of inference is lagging. Environmental impact studies for bats, largely guided by regulatory requirement, are generally limited to asking whether a species is present or absent and may consider a rudimentary evaluation of relative activity levels such as comparing the total number of passes recorded or the average number of passes per night between sites [5]. Additionally, reliable species identification requires the analysis of a single call type, the search phase call emitted when a bat scans its environment [6, 7]. While comparing acoustic activity levels can indicate the relative interest placed by bats in a site, it does not help understand how the bats are using the site. Untapped information contained within echolocation recordings beyond these simple parameters may help determine how bats use or perceive a particular site, which is critical to developing and applying effective conservation measures [8].

Terminal phase echolocation calls or ‘feeding buzzes’, sequences of short pulses emitted in rapid succession, are produced by bats when they home in to and capture prey [9, 10]. Feeding buzzes, therefore, have the capacity to act as an index of foraging activity and indeed, many studies have tallied feeding buzzes to quantify bat foraging activity and test hypotheses to explain local bat activity levels [11, 12]. Quantifying feeding buzzes in bat recordings should provide a wealth of additional information that will be valuable in evaluating bat habitat. For example, comparing the relative proportion of feeding buzzes per bat pass across sites could help in assessing how bats use a given site [11]. Patriquin and Barclay [13] found that foraging activity was a better indicator of habitat type for *Myotis* species compared to the number of acoustic bat passes. Changes in feeding buzz patterns may also serve as indicators of disturbance or habitat alterations caused by human activities. When the goal is to identify areas near critical habitat features such as roosts or hibernacula, identifying the primary activity type at the site (foraging, social etc.) may be invaluable for deducing the presence of such features and for directing survey efforts. Thus, feeding buzzes have the potential to provide valuable insights when identifying areas for prioritizing conservation efforts.

Despite the potential value in quantifying feeding buzzes from bat survey recordings, their inclusion is mostly limited to hypothesis testing in scientific studies [12, 14, 15], likely owing to past difficulty in automating their detection in recordings. Most available automated classifiers for bat echolocation data focus on species identification and are therefore optimized to detect search phase calls that are usually the loudest signals recorded. However, compared to search phase calls, feeding buzz pulses are generally characterized by much shorter durations and are emitted at much higher rates (> 100 pulses/s) [16]. Bats also increase the directionality of feeding buzz signals [17], which often leads to a reduced signal-to-noise ratio (SNR: strength of the call relative to the background noise) in recordings. These features make it much more difficult for current software to detect feeding buzzes and is why they have mainly been quantified through visual examination of call spectrograms [15, 18, 19] or by listening to time-expanded recordings [12]. Fortunately, recognition of the importance of automating the detection of feeding buzzes has gained momentum. Some studies have partially automated the detection of feeding buzzes by applying thresholds to the pulse production rate obtained from a signal detection algorithm [20, 21]. While this approach can reduce the time needed for manual identification of feeding buzzes, extensive manual vetting may still be needed. Additionally, signal detection algorithms may still miss weaker buzz signals potentially resulting in a considerable number of false negatives. More recently, Roemer et al. [22] used a random forest algorithm to develop a classifier of bat call sonotypes, which included feeding buzzes, from parameters extracted using the Tadarida R package, and a feeding buzz detection tool is in development for the automated classification software Sonobat (Arcata, CA, USA). The former is free to use while the latter requires software purchase. Making diverse acoustic classification tools accessible is crucial for enhancing widespread adoption of comprehensive data extraction methods, harnessing the full potential of acoustic data to refine inferences, and ultimately advancing conservation outcomes.

I tested a new method to optimize detection of feeding buzzes in echolocation recordings using a freely available acoustic detection algorithm from the “Bioacoustics” package in the R environment [23]. Based on the signals detected, testing three modelling frameworks (linear discriminant analysis, random forest, and artificial neural networks), I developed a model to automate the classification of recordings containing feeding buzzes. I then developed “buzzfindr”, a user-friendly function in R that integrates the automatic detection and classification of feeding buzzes which I tested and evaluated on a new set of recordings.

## Materials and methods

### Ethics statement

No ethical approvals were required for this work as acoustic recordings were passively obtained without disturbing any animals.

### Training data

**Fig 1:**
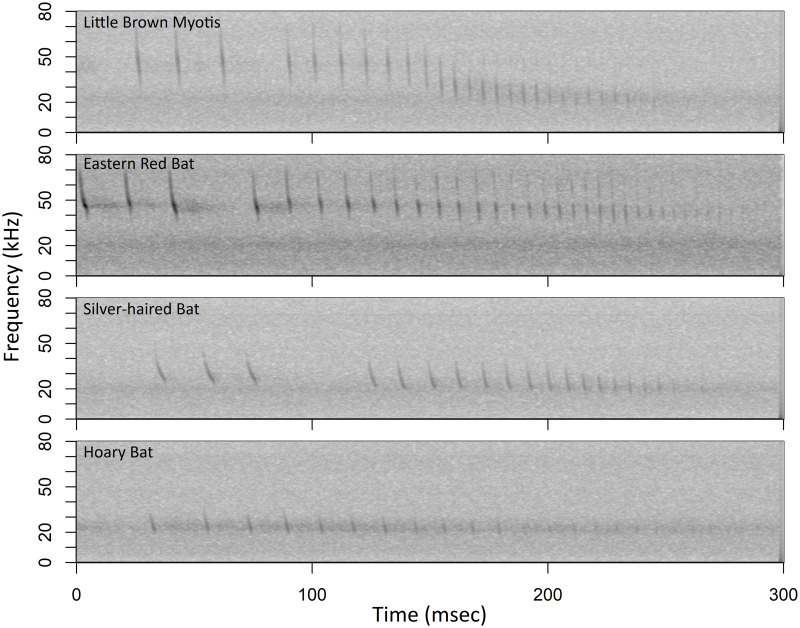
Feeding buzzes from four bat species confirmed in data used to train and test the classifier. Example spectrograms of feeding buzzes from four bat species confirmed in data used to train and test the classifier. For the first three species (Little Brown Myotis, Eastern Red Bat, Silver-haired Bat), the initial three pulses preceding the buzz belong to the approach phase.

I compiled data to train the feeding buzz classifier from recordings of bats made at five locations throughout Ontario, Canada, with three different recording devices over three separate years (Table 1). The three devices were the Song Meter SM2BAT+ coupled with an SMX-US or SMX-UT microphone, the Song Meter SM4BAT–FS coupled with a SMM–U2 microphone, and the Song Meter Mini Bat with an integrated microphone, all manufactured by Wildlife Acoustics Inc. Variation among recorder types and microphones leads to differences in the amplitude, spectral characteristics, and noise profiles of their recordings which can also lead to differences in their ability to sample bat activity [24]. As such, data from more than one device were used to improve the generalizability of the classifier and to test whether recorder type affected classifier accuracy. Recording devices were deployed by biologists conducting environmental impact studies across northern Ontario from 2013–2021, with a single device type deployed at each project. Due to data sharing agreements, the precise locations of the recording sites cannot be provided. Recorders were deployed along forest edges beside wetlands or open shrublands. Forest stands were mainly mixed, dominated by Black Spruce *(Picea mariana)*, Trembling Aspen (*Populus tremuloides*) and White Birch (*Betula papyrifera*). Microphones were positioned 3–4m above ground, facing open habitat away from physical obstructions and prevailing winds. Recordings were made with a 384 kHz sampling frequency. Detailed recorder settings are provided in the S1 File. I manually classified all recordings used in training and testing the classifier to species. Recordings were by the following species or species guilds: Silver-haired Bat (*Lasionycteris noctivagans)*, Silver-haired Bat or Big Brown Bat (*Eptesicus fuscus*), Hoary Bat (*Lasiurus cinereus* or *Aeorestes cinereus*), Eastern Red Bat (*Lasiurus borealis*), Little Brown Myotis (*Myotis lucifugus*), and unknown species of the genus Myotis (an example feeding buzz from each confirmed species is provided in Fig 1; S2 File contains detailed species counts for training and testing data). I compiled recordings of feeding buzzes and recordings without feeding buzzes by visually inspecting spectrograms of the recordings with program Audacity 2.4.2 (Boston, Ma). I selected calls irrespective of their signal-to-noise-ratio but excluded recordings containing anomalous microphone interferences. I highlighted each observed feeding buzz from the approximate start of the buzz to just after the last perceived pulse in the buzz sequence and saved the highlighted sequence as a separate file. For each new buzz file, I used Audacity’s labelling function to label the start and end of all pulses in the buzz sequence and exported the series of pulse start and end times as a txt file. For recordings without buzzes, I used the entire unlabeled file.

**Table 1:** Summary of data used to train the feeding buzz classifier.

Site	Date Range	Recording Device	Microphone	Number of Devices	High-Frequency		Low-Frequency	
					Buzz	No Buzz	Buzz	No Buzz
1	21–26 May, 2013	SM2BAT+	SMX–US	1	3	25	13	9
2	12–23 September, 2013	SM2BAT+	SMX–UT	1	21	26	0	0
3	1–30 June, 2021	SM4BAT	SMM–U2	3	34	36	27	41
4	23–17 July, 2020	SM4BAT	SMM–U2	1	21	30	0	0
5	5–6 July, 2021	Mini Bat	Integrated	2	13	15	26	24

The table above is a summary of the data used to train the feeding buzz classifier. Data were collected from sites across Ontario, Canada.

### Data extraction

Automated signal detection within the recordings was achieved using the ‘threshold_detection’ function of the ‘bioacoustics’ package in R [23]. This function is optimized for detecting ultrasonic signals within recordings and is highly flexible in its parameterization. Its detection algorithm is adapted from that by Scott [25] that estimates and subtracts the noise floor independently for each frequency component of the Fast Fourier Transform (FFT) spectrum using past values of the recording. When the algorithm is applied to the full frequency range of interest, as would be required for detecting and classifying search-phase calls, it does not detect feeding buzz pulses due to their short duration and often low bandwidth and SNR. However, I was able to extract the subtle buzz signals with the algorithm by sequentially scanning narrow frequency bands using bandpass filters. I used seven frequency bands, each band spanned 5 kHz with the lowest frequency band ranging from 15–25 kHz and the highest one ranging from 45–50 kHz. I identified the levels of the detection function parameters that maximized the detection of buzz signals while minimizing noise detections. For each of 10 parameters, I selected three levels that allowed a suitable exploration across the range of possible values. I then tested every set of 10 parameters across all 69,984 possible combinations of the parameter levels (S3 File). The optimal combination specified a minimum signal duration (min_dur) of 0.2 msec, a minimum time window between audio events (min_TBE) of 2 msec, an overlap between consecutive FFT windows (FFT_overlap) of 87%, an SNR threshold (SNR_thr) of 4 dB, a maximum duration before background noise monitoring is resumed (duration_thr) of 80 msec, an angle threshold to designate the end of a detected signal (angle_thr) of 40°, an amplitude threshold for the start and end of the detected signal (start_thr and end_thr resprectively) of 20 dB, a background noise estimation window (NWS) of 20 msec, and a threshold parameter (threshold) of 5 dB. The ‘threshold’ parameter controls the sensitivity of the spectral peak detection algorithm relative to the SNR. All other parameters were conserved at their default values. The SNR of extracted signals calculated by the detection algorithm ranged between 5.3–26.4 dB for buzz pulses and 1.6–22.3 dB for non-buzz signals. These values are specific to the detection window and parameters used.

From each signal detected by the algorithm (echolocation or noise segment), I extracted the following parameters: The duration, the SNR, the slope of the time-frequency trend, the smoothness which is a measure of the variation in the time-frequency trend of the call, and the interpulse interval (IPI) which was taken as the time between the beginning of two consecutive signals. I used a moving window spanning four consecutive detection events to calculate variables describing the variation and change in the call-specific parameters. Specifically, I regressed IPI over the signal number (1,2,3 etc.) in the sequence and extracted the slope coefficient (IPIslope) and the intercept (IPIint) of the regression. I also calculated the minimum and maximum IPI (IPImin, IPImax), the average IPI (IPIavg), and the standard deviation, variance and Shannon entropy of the IPI (IPIsd, IPIvar, IPIshannon). With respect to SNR, I extracted the adjusted rsquared for the regression of the signal SNR over the signal number (1,2,3 etc.; SNRr), as well as the minimum, maximum and average SNR (SNRmin, SNRmax, SNRavg), and the standard deviation and variance of the SNR (SNRsd, SNRvar). Finally, with respect to the call slope, I calculated the average slope (slopeavg), the minimum slope (slopemin), and the standard deviation of the slope (slopesd). I also calculated the average signal duration (duravg) and its standard deviation (dursd). Finally, I calculated the average of the smoothness parameter (smoothavg) and its variance (smoothvar).

### Model development

Following the calculation of variables, I removed 146 extreme outliers in the non-buzz dataset (SNRvar > 3000 and IPIshannon < -10) and scaled all predictor variables. I used a Pearson correlations analysis and a Principal Components Analysis (R function *prcomp*) to identify highly correlated variables (r > 0.6) in the dataset and prioritized those with the greatest explanatory power and with a low correlation between each other. Variables loading along the first principal component (PC1) axis drove the separation between buzz and non-buzz signals so variables with the strongest loading along this axis were those retained for the model. To avoid overfitting the model and ensure balanced representation of all signal variants within and between each group (buzz or non-buzz), I divided the PC1 scores of each group into five equal quantiles and randomly subsetted 150 PC1 values from each quantile. The final dataset of 1500 signals comprised the rows from the original data associated with the subsetted PC1 values. The final dataset met established minimum machine learning sample size criteria [26, 27]. I then divided the subsetted data into testing and training sets with 80% of the data assigned to the training set, and 20% to the testing set. I modelled the classification of buzz and non-buzz signals using three classification methods commonly used in machine learning [27, 28]: Linear discriminant analysis (LDA; function *lda* of R package MASS), random forest (RF; function *randomForest* of R package randomForest), and artificial neural networks (ANN; function *neuralnet* of R package neuralnet). I compared the classification accuracy (i.e. proportion of accurately classified passes) of each model type after obtaining predicted values for the models with R function *predict* at classification thresholds of 0.5–1.0. Random forest (RF) and ANN gave the highest accuracies (see results section) so I built final models from the entire balanced dataset with both methods and tested each model on a new dataset.

### Model testing

I combined the data extraction procedure and classification into a single R function called “buzzfindr”. I tested two instances of buzzfindr, one with the RF model and one with the ANN model, on a set of 889 recordings from nine new sites across Ontario, Canada (Table 2). Habitat and equipment deployment conditions were identical to those used for the training data at all sites except 3 and 9 which were in urban parks in southern Ontario along deciduous woodlot edges. Each third of the test recordings was from a different recording device model and species frequency group (high-frequency / low-frequency) and buzz class (contained a buzz / lacked a buzz) were equally distributed within each device type. I evaluated different performance metrics for the models with function *confusionMatrix* of R package caret to determine their suitability including their sensitivity, specificity, and final balanced accuracy [29]. Sensitivity also termed ‘Recall’ is the model’s ability to predict true buzzes and is measured as *TP*/(*TP* + *FN*) where TP is the number of true positives and FN, the number of false negatives. Specificity describes the model’s ability to predict true non-buzzes and is calculated as *TN*/(*TP* + *FP* + *TN* + *FN*) where FP and TN are the number of false-positives and true negatives respectively [29]. I also compared the receiver operating characteristic curves (ROC) of both models to assess the trade-off between sensitivity and specificity of each. Both models gave nearly identical ROCs but RF provided better control over the false positive rate (see results) so it was retained as the final model used in buzzfindr.

**Table 2:** Summary of data used to test the feeding buzz classifier.

Site	Date Range	Recording Device	Microphone Model	Number of Devices	High-Frequency		Low-Frequency	
					Buzz	No Buzz	Buzz	No Buzz
1	29–30 May, 2013	SM2BAT+	SMX–US	2	43	42	71	75
2	20 June–16 July, 2016	SM2BAT+	SMX–UT	6	27	27	-	-
3	31 May–1 June, 2021	SM2BAT+	SMX–U1	1	-	1	7	6
4	5–24 June, 2021	SM4BAT	SMM–U2	3	19	21	65	63
5	28 August–5 September, 2021	SM4BAT	SMM–U2	1	46	44	4	7
6	15 July–18 August, 2022	SM4BAT	SMM–U2	8	23	23	-	-
7	15 June–31 July, 2021	Mini Bat	Integrated	9	12	12	32	32
8	16 June–19 July, 2021	Mini Bat	Integrated	13	46	44	16	16
9	13 June–5 July, 2022	Mini Bat	Integrated	3	9	10	23	23

The table above is a summary of the data used to test the feeding buzz classifier. Data were collected across Ontario, Canada.

To determine if classifier accuracy was biased toward a specific recorder type or species frequency group, I used a logistic regression to compare the probability of correctly classifying a buzz against recording device type, frequency group, and their interaction. Within frequency group, low-frequency bats were species that emit calls with a minimum frequency below 30 kHz (Hoary Bat, Silber-haired Bat and Big Brown Bat) while high-frequency bats were those whose minimum frequency is above 30kHz (species of the genus *Myotis* and Eastern Red Bat). In addition to examining model performance, the practicality of the tested method will also depend on the speed at which recordings are processed. Therefore, I also measured buzzfindr’s average processing time per recording on two computers with contrasting processing capabilities: a Dell Latitude laptop with a i5–2520M CPU and 8Gb of RAM and a Dell Precision 7680 with a i7–13850HX CPU and 32Gb of RAM.

## Results

### Model development

I identified variables with the greatest explanatory power to include in the buzz detection model by running a PCA on 21 parameters extracted from 1,231 detection event sequences from recordings containing a buzz and 26,992 detection event sequences from recordings that did not contain a buzz (S4 File). The first dimension of the PCA accounted for 23.0% of the variance in the data. The variables that loaded most strongly along this axis and thus, those used in building the model were the standard deviation of the inter-pulse interval (IPIsd), the average duration of the detected signal (duravg), and the average SNR of the detected signal (SNRavg). I evaluated the accuracy of the classifier at classification decision thresholds ranging from 0.5 to 1 in increments of 0.05. When using the training data to test the accuracy of a model built with the three different modelling procedures, the highest classification accuracies obtained were 96% for LDA at a 0.6 decision threshold, 100% for RF at a 0.7 decision threshold, and 100% for ANN at a 0.85 decision threshold. The latter two methods gave accuracies of 98% and 100% respectively at a 0.8 threshold and were considered equivalent (S5 File).

### Model testing

**Fig 2:**
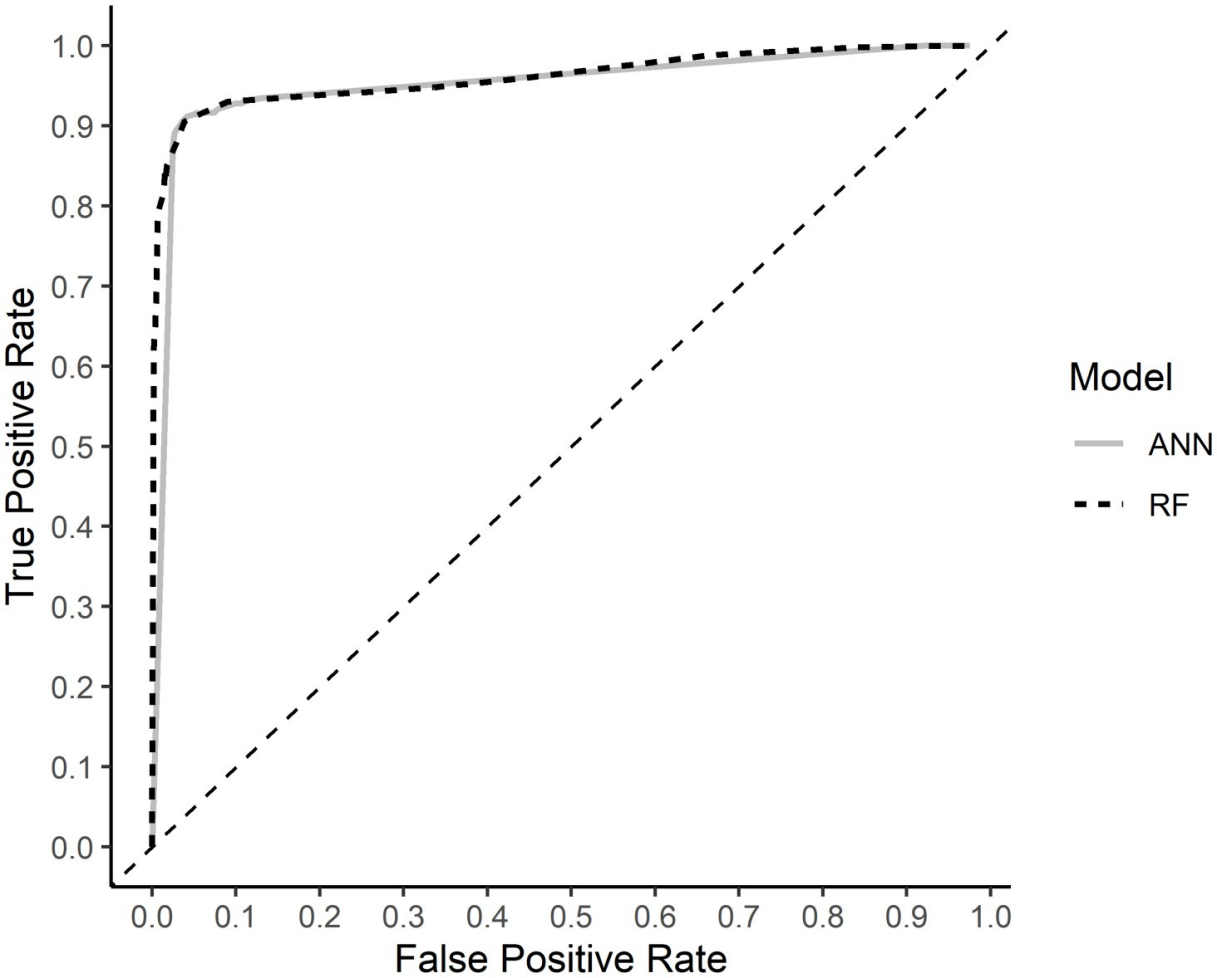
Receiver operating characteristic curves (ROC) of a random forest (RF) and an artificial neural network (ANN) buzz classifier. ROC curves were generated by running both classifiers on a new dataset of recordings and measuring their sensitivity (true positive rate) and specificity (*false positive rate* = 1—*specificity*) across a range of classification thresholds.

**Fig 3:**
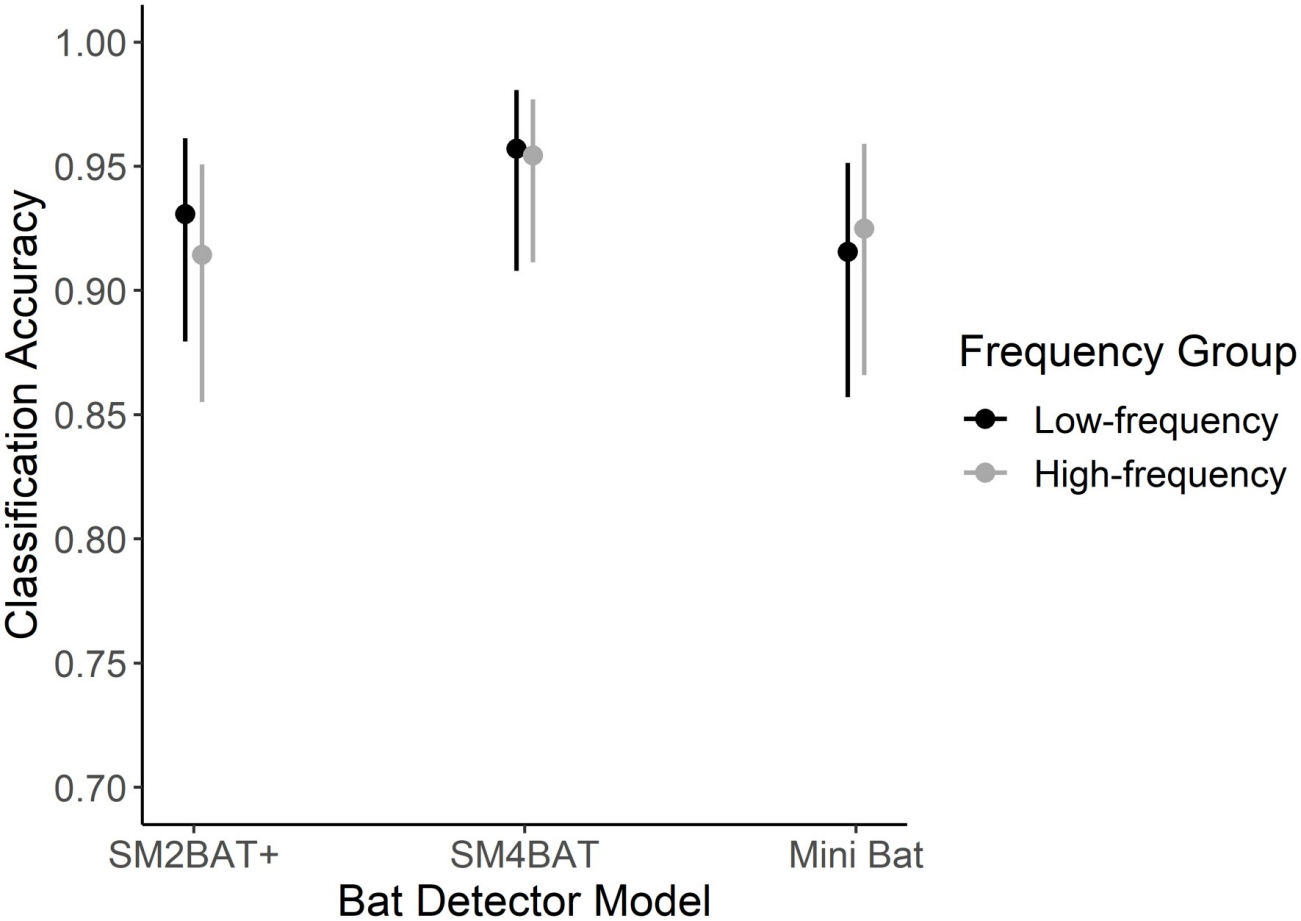
Classifier accuracy by recorder type and species frequency group. Comparison of the accuracy of the classifier to correctly classify recordings from three different bat recorder models and two species frequency groups as containing or not containing a buzz. Accuracy is reported as the mean probability of correct classification ± 95% confidence intervals.

Two versions of buzzfindr, one with the RF model and one with the ANN model were iteratively tested on a new dataset of 889 recordings at incremental classification decision thresholds. The resulting ROC curves were nearly identical but the RF model provided greater control of the false-positive rate (reached zero at much lower thresholds) compared to the ANN model (Fig 2). The final RF model performed with a sensitivity of 90.5%, a specificity of 96.2%, and an overall mean accuracy of 93.4% (95% CI: 91.5–94.9%) given a classification decision threshold of 0.8. The average processing time for the slower computer model (Dell Latitude) was 1.31 seconds/file with sequential processing and 0.55 seconds/file with parallel processing using four logical processors. The faster computer model (Dell Precision) gave speeds of 0.47 seconds/file with sequential processing and 0.06 seconds/file with parallel processing over eight logical processors. The average file length was 4.92 seconds. Recordings containing buzzes or many call events were processed more slowly than those with few call events. A logistic regression testing the effect of recorder type and species frequency group on the accuracy of the classifier showed that buzzfindr’s overall accuracy remained constant across recorder model and species frequency group (Fig 3; S6 File).

## Discussion

The principal drawback to quantifying feeding buzzes has always been the time and effort requirements involved. Although other partial or complete automated methods exist such as applying user-set thresholds to specific acoustic parameters or using machine-learning models, the signal detection process they employ generally relies on applying an amplitude threshold over the entire spectral area of interest within a moving window [20–22]. This can lead to a higher noise floor which lowers the SNR causing weaker buzz signals to be missed. Focusing an acoustic signal detection algorithm on narrow frequency bands resulted in successful detection of weak buzz events, likely by lowering the noise floor of the frame being analyzed. Additionally, sufficient information could be extracted from these detections to classify buzzes with a high degree of accuracy.

Bat acoustic recordings have the potential to reveal more to us than simply the number of times a given species was recorded by the microphone. Integrating new sources of information in the general analytical framework of bat acoustic data could have far-reaching benefits on bat conservation and research. For example, the rate of feeding buzz production (e.g. buzzes/pass) can be used as a response variable in multivariate models to understand its relationship to habitat and other extrinsic environmental factors. Additionally, the number of detected buzzes could be easily included as a covariate representing site-specific prey abundance in occupancy models. In the context of anthropogenic habitat disturbances, feeding buzz rate could be used as an indicator for estimating potential impacts on foraging habitat, identifying the geographic reach of existing disturbances on foraging behavior, evaluating the success of post-disturbance remediation of foraging habitat, and identifying suitable lands to protect as compensatory foraging habitat when disturbances cannot be sufficiently mitigated. Given the potential benefits of adding this single new source of information to the bat researcher’s analytical toolbox, more effort should be directed toward identifying additional sources of information in acoustic data and facilitating their extraction. For example, there is evidence that bat echolocation encodes information about the emitter’s sex, age, reproductive and body condition [30], group membership [31, 32], and possibly the location of roosts and foraging sites [33, 34]. Temporal and spectral attributes of recorded calls may also help infer a bat’s position relative to the microphone and clutter [34]. Finally, social calls may also serve as potential indices of bat behavior, health, sex and reproductive status [33, 35, 36]. Given the pool of potential clues to exploit in acoustic bat recordings, an extensive array of acoustic indices could eventually be derived and integrated into a single statistical framework to answer a wide range of questions on behavior and habitat use.

The structure of feeding buzzes is relatively simple and consistent [16], making their identification possible from simple parameters related to temporal patterns of pulse production (signal duration and variation in the repetition rate). The average SNR also emerged as a significant predictor. This is likely because the signal detection algorithm was tuned to identify faint signals. Consequently, it not only identifies weaker buzzes but also detects any non-buzz spectral peak above the parameter-defined threshold. Average SNR likely plays a crucial role in parsing out genuine bat signals from random noise. One drawback to the simplicity of the method used is that buzzfindr may confuse closely spaced short-duration broadband noises (which can result from equipment interference or environmental sources) as buzzes, which can increase the rate of false positives. To correct for this, it could be possible to implement a filter that scans a wider range of narrower frequency bands (1kHz) to identify and remove detections that span frequencies falling outside the possible range for a buzz. Other ways of improving accuracy in detecting buzzes in noisy recordings could include only processing files known to contain echolocation calls or applying additional post-classification decision rules. To address this, I have included two user-specified arguments in buzzfindr, one that performs an initial scan of the recordings for echolocation-like signals and the other that applies a voting procedure on the initial classifications before making a final decision. Another limitation stems from the classifier’s dependence on accurately detecting individual buzz pulses. Feeding buzzes are highly susceptible to acoustic scattering, and this can limit the algorithm’s ability to perceive the pulses that comprise them. Given that degraded buzzes are still often visually discernable in the spectrogram, greater accuracy in future feeding buzz classifiers could be achieved by leveraging deep-learning tools such as convolutional neural networks to train classifiers on images of buzz spectrograms. Although buzzfindr was trained on calls from only four confirmed species covering a restricted geographic range, its classification does not depend on frequency so species effects related to frequency should be minimal. This is supported by it demonstrating equivalent accuracies between species frequency groups (Fig 3). Additionally, the frequency range of interest can be adjusted to detect buzzes beyond the training range and training can always be expanded to new species. Finally, while the classifier’s performance was unaffected by device type suggesting it may be robust to differences between different recorders, all devices used were by the same manufacturer and the same settings were applied to all devices of a given type. Thus, classifier performance may still vary with device type or device settings. This potential bias could be mitigated by adjusting the sensitivity of the detection algorithm and further testing is needed to understand its implications.

Another consideration is that emission of a buzz does not always indicate that the bat was successful in capturing its prey [37]. Successful prey capture can be identified by a pause in calling immediately following the buzz which indicates the bat is consuming its prey [38]. It could be possible to implement a procedure in automated buzz recognition that identifies successful captures based on the post-buzz pause. However, this likely requires accurate identification of the end of the buzz and the onset of the subsequent call sequence, and the method described here does not detect all buzz pulses in a buzz sequence, nor is it optimized to identify search phase calls. Incidentally, since buzzfindr cannot identify precise start and end times of a buzz, it identifies multiple buzzes within a single recording via a time threshold between positive buzz detections. The rate of buzz production at a site may also depend on the type of prey at the site. For example, many small prey will elicit a higher buzz rate compared to fewer large prey. The predation strategy of the bat, or conversely the anti-predator strategy of the insect, are also likely to influence the rate of buzz production. Some gleaning bats stop vocalizing and omit the feeding buzz just before capturing prey [39, 40]. Many insects can hear high frequencies and have evolved antipredator countermeasures in response to approaching bats [40]. By impacting predation success, these evasive behaviours may influence the rate of buzz production. Finally, some bat species are attracted to the feeding buzzes of conspecifics [41–43] which may also influence the rate of buzzing at a site. While the rate of buzzing should still be a reliable indicator of foraging activity, considering habitat characteristics, as well as the community structures of local bats and their insect prey could help refine the inferences gained from analyzing habitat use by bats from feeding buzzes.

## Conclusion

For over two decades, inferences drawn from acoustic bat data have relied on the same metrics, primarily due to the lack of tools facilitating the acquisition of other ecological indicators besides species [6, 44, 45]. Here I describe a free tool for quantifying feeding buzzes that can be easily implemented in any bat acoustic analytical workflow. Despite buzzfindr’s potential limitations, it promises significant time savings compared to less automated approaches, even when accompanied by post-hoc manual vetting which is still best practice when using automated classifiers. Moreover, its low processing needs and ease of accessibility and implementation hold the potential to foster a broader recognition of the value of examining feeding buzzes when interpreting acoustic bat data. Tools such as buzzfindr will help enrich the inferences obtained by researchers and conservation practitioners to better inform bat conservation and management.

## Supporting information

S1 File Recorder setting used in data collection. Settings used in the deployment of each acoustic recorder model used to record bat echolocation calls.
(PDF)

S2 File Data species composition. Species classification of bat call sequences used to train and test the buzz classifier. Passes were classified to species manually. Mylu = *Myotis lucifugus*, Labo = *Lasiurus borealis*, Lano = *Lasionycteris noctivagans*, Epfu = *Eptesicus fuscus*, Laci = *Lasiurus cinereus*.
(PDF)

S3 File Number of detections by the signal detection algorithm of buzz and non-buzz signals for 69,984 detection parameter combinations. I identified the levels of the detection algorithm parameters that maximized the detection of buzz signals while minimizing noise detections. For each of 10 parameters, I selected three levels that allowed a suitable exploration across the range of possible values and tested every set of 10 parameters across all 69,984 possible combinations of the parameter levels.
(PDF)

S4 File Biplot (axes 1 and 2) from a principal components analysis of variables calculated from buzz and non-buzz (control) echolocation calls. The following variables were calculated for each sequence of four consecutive signals from the signal inter-pulse interval (IPI), signal duration (dur), signal SNR (SNR) and signal smoothness (smooth): IPIslope = slope from a regression on IPI, IPIint = intercept from a regression on IPI, IPImin = minimum IPI, IPImax = maximum IPI, IPIavg = average IPI, IPIsd = standard deviation of the IPI, IPIvar = variance of the IPI, IPIshannon = Shannon entropy of the IPI, SNRr = adjusted rsquared for the regression on the signal SNR, SNRmin = minimum SNR, SNRmax = maximum SNR, SNRavg = average SNR, SNRsd = standard deviation of the SNR, SNRvar = variance of the SNR, slopeavg = average slope, slopemin = minimum slope, slopesd = standard deviation of the slope, duravg = average call duration, dursd = standard deviation of the call duration, smoothavg = average of the smoothness parameter, smoothvar = variance of the smoothness parameter.
(PDF)

S5 File Accuracy of three modelling methods in detecting feeding buzzes in test-data. The accuracy in detecting feeding buzzes in test-data (i.e. proportion of accurately classified passes) was examined for three classification methods (LDA: Linear discriminant analysis; RF: Random forests; ANN: Artificial neural networks) at incremental detection threshold levels.
(PDF)

S6 File Effect of recorder type and species frequency group on classifier accuracy. Results from a logistic regression testing the effect of recorder type (SM2BAT+, SM4BAT, Bat Mini) and species frequency group (High-frequency, Low-frequency) on the accuracy of the classifier.
(PDF)
