# Supplementary material for: Buzzfindr: Automating the detection of feeding buzzes in bat echolocation recordings
Source: PLoS One. 2024 Aug 20;19(8):e0306063. doi: 10.1371/journal.pone.0306063 (PMC11335113; doi:10.1371/journal.pone.0306063)
Supplement: S1 File — Settings used in the deployment of each acoustic recorder model used to record bat echolocation calls. (PDF) [file pone.0306063.s001.pdf]

S1 File. Recorder setting used in data collection. Settings used in the deployment of each acoustic recorder model used to record bat echolocation calls.

Recorder model	SM2BAT+		SM2BAT+		Recorder model	SM4BAT+
Microphone	SMX-US		SMX-UT		Microphone	SMM-U2
**Analog settings**						
2.5 Volt Bias	OFF		OFF			
HPF	1000 Hz		1000 Hz			
GAIN	48 dB		36 dB			
**Audio Settings**					**Audio Settings**	
Sample rate	384 kHz		384 kHz		Gain	12 dB
Channel	mono-R (right)		mono-R (right)		16k High Filter	OFF
Compression	None (WAV)		None (WAV)		Sample rate	384 kHz
Gain left	+0.0 dB		+0.0 dB		Min Duration	1.5 ms
Gain right	+0.0 dB		+0.0 dB		Max Duration	none
Dig HPF left	fs/32 (12 kHz)		fs/32 (12 kHz)		Min Trig Freq	14 kHz
Dig HPF right	fs/32 (12 kHz)		fs/32 (12 kHz)		Trigger Level	12 dB
Dig LPF left	Off/disabled		Off/disabled		Trigger Window	2s
Dig LPF right	Off/disabled		Off/disabled		Max Length	5s
Trg Lvl left	18 SNR (+18dB)		18 SNR (+18dB)		Compression	none
Trg Lvl right	18 SNR (+18dB)		18 SNR (+18dB)		**Delayed Start**	disabled
Trg Win left	2.0s		2.0s		**LED Indicator**	always
Trg Win right	2.0s		2.0s			
Trg Max Length	5s		5s			
Div Ratio	16		16			
**Scheduled Recording**					**Scheduled Recording**	
Schedule	Advanced		Advanced		Do Not Sleep	no
	AT SSET -00:30:00		AT SSET -00:30:00		Schedule mode	daily
	DO		DO		Schedule	Advanced
	RECORD 12:00:00		RECORD 12:00:00			START: set - 00:30
	UNTSRIS +00:30:00		UNTSRIS +00:30:00			DUTY: always
	GOTO LINE 01 20X		GOTO LINE 01 20X			END: rise + 00:30
**Other**					**Other**	
Solar calculation	Sunrise/Sunset		Sunrise/Sunset		Solar Calculation	Sunrise/set

Recorder model	Bat Mini
**Ultrasonic Settings**	
Mode	Full Spectrum
Sample Rate	384 kHz
Min Trigger Freq.	14 kHz
Max Recording Length	5 seconds
Trigger Window	2 seconds
Save noise files?	Toggled OFF
Left Channel Gain	12 dB
**Scheduled Recording**	
Mode	Ultrasonic
Start Date	Current date
Start Time	Set – 00:30
Time Duty Cycle	Always
End Time	Rise + 00:30
End date	Current day + 40 days
