# Supplementary material for: Buzzfindr: Automating the detection of feeding buzzes in bat echolocation recordings
Source: PLoS One. 2024 Aug 20;19(8):e0306063. doi: 10.1371/journal.pone.0306063 (PMC11335113; doi:10.1371/journal.pone.0306063)
Supplement: S2 File — Species classification of bat call sequences used to train and test the buzz classifier. Passes were classified to species manually. Mylu = Myotis lucifugus, Labo = Lasiurus borealis, Lano = Lasionycteris noctivagans, Epfu = Eptesicus fuscus, Laci = Lasiurus cinereus. (PDF) [file pone.0306063.s002.pdf]

S2 File. Data species composition. Species classification of bat call sequences used to train and test the buzz classifier. Passes were classified to species manually. Mylu = *Myotis lucifugus*, Labo = *Lasiurus borealis*, Lano = *Lasionycteris noctivagans*, Epfu = *Eptesicus fuscus*, Laci = *Lasiurus cinereus*.

Modeling Data – Buzz

Site	Unknown Myotis	Mylu	Labo	Lano	Lano/Epfu	Laci
1	3				13	
2	21					
3	1		20			
4	33	1		19	2	6
5	1	12		20	2	4

Modeling Data - Not a Buzz

Site	Unknown Myotis	Mylu	Labo	Lano	Lano/Epfu	Laci
1	22	3			8	1
2	25	1				
3			30			
4	35	1		11	25	5
5	15			5	11	8

Training Data – Buzz

Site	Unknown Myotis	Mylu	Labo	Lano	Lano/Epfu	Laci
1	31	12		2	69	
2	19	6	2			
3					7	
4	17	2		6	53	6
5	43	3		1	3	
6			23			
7	12			25	7	
8	39	7		2	12	2
9			9	1	22	

Training Data - No Buzz

Site	Unknown Myotis	Mylu	Labo	Lano	Lano/Epfu	Laci
1	26	16			75	
2	19	6	2			
3	1				4	2
4	19	2		18	39	6
5	36	7		3	4	1
6			23			
7	12			14	18	
8	37	7		2	7	7
9			10	1	22	
