# Supplementary material for: Buzzfindr: Automating the detection of feeding buzzes in bat echolocation recordings
Source: PLoS One. 2024 Aug 20;19(8):e0306063. doi: 10.1371/journal.pone.0306063 (PMC11335113; doi:10.1371/journal.pone.0306063)
Supplement: S3 File — I identified the levels of the detection algorithm parameters that maximized the detection of buzz signals while minimizing noise detections. For each of 10 parameters, I selected three levels that allowed a suitable exploration across the range of possible values and tested every set of 10 parameters across all 69,984 possible combinations of the parameter levels. (PDF) [file pone.0306063.s003.pdf]

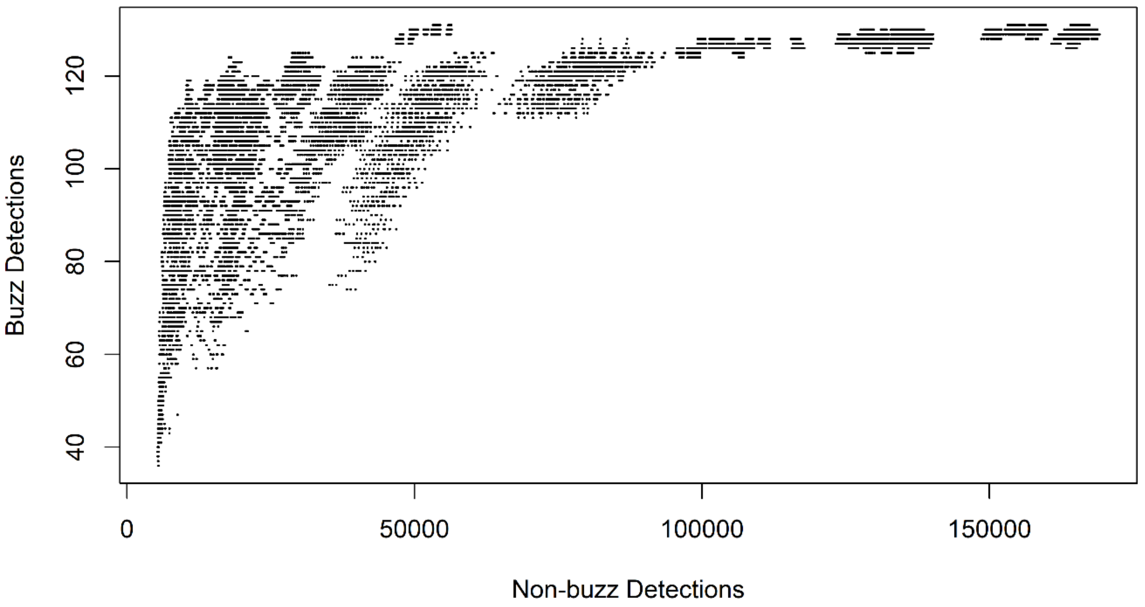

S3 File. Number of detections by the signal detection algorithm of buzz and non-buzz signals for 69,984 detection parameter combinations.
