# Supplementary material for: Buzzfindr: Automating the detection of feeding buzzes in bat echolocation recordings
Source: PLoS One. 2024 Aug 20;19(8):e0306063. doi: 10.1371/journal.pone.0306063 (PMC11335113; doi:10.1371/journal.pone.0306063)
Supplement: S4 File — The following variables were calculated for each sequence of four consecutive signals from the signal inter-pulse interval (IPI), signal duration (dur), signal SNR (SNR) and signal smoothness (smooth): IPIslope = slope from a regression on IPI, IPIint = intercept from a regression on IPI, IPImin = minimum IPI, IPImax = maximum IPI, IPIavg = average IPI, IPIsd = standard deviation of the IPI, IPIvar = variance of the IPI, IPIshannon = Shannon entropy of the IPI, SNRr = adjusted rsquared for the regression on the signal SNR, SNRmin = minimum SNR, SNRmax = maximum SNR, SNRavg = average SNR, SNRsd = standard deviation of the SNR, SNRvar = variance of the SNR, slopeavg = average slope, slopemin = minimum slope, slopesd = standard deviation of the slope, duravg = average call duration, dursd = standard deviation of the call duration, smoothavg = average of the smoothness parameter, smoothvar = variance of the smoothness parameter. (PDF) [file pone.0306063.s004.pdf]

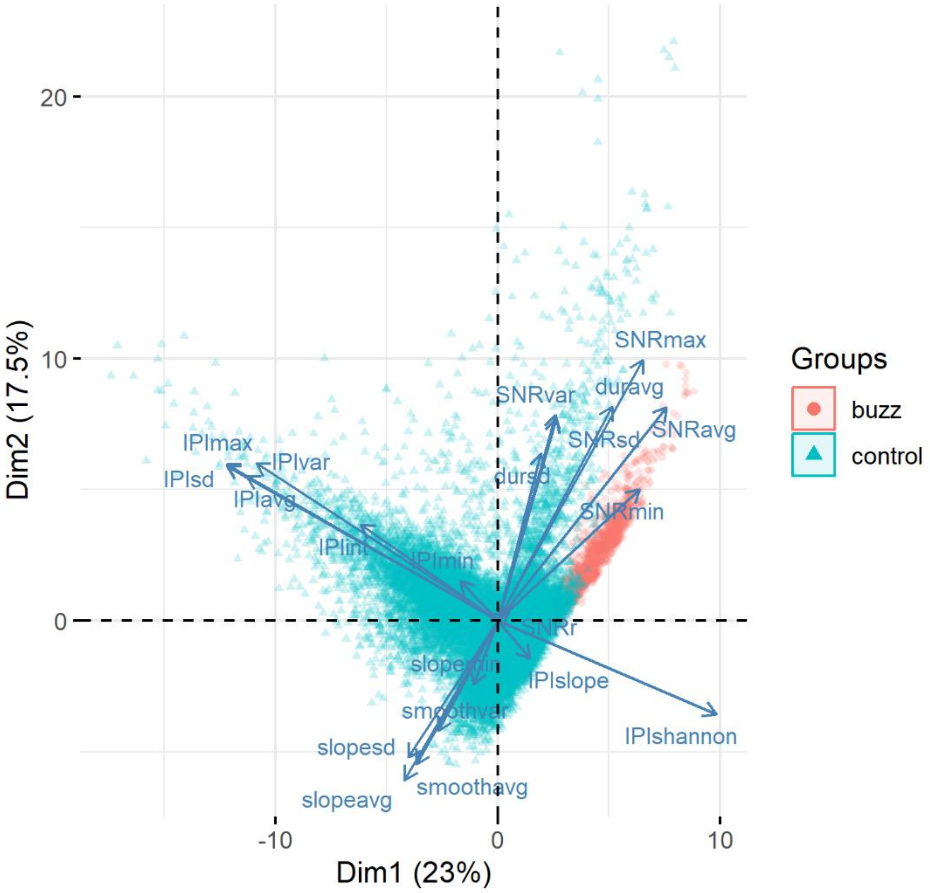

File S4. Biplot (axes 1 and 2) from a principal components analysis of variables calculated from buzz and non-buzz (control) echolocation calls. The following variables were calculated for each sequence of four consecutive signals from the signal inter-pulse interval (IPI), signal duration (dur), signal SNR (SNR) and signal smoothness (smooth): IPIslope = slope from a regression on IPI, IPIint = intercept from a regression on IPI, IPImin = minimum IPI, IPImax = maximum IPI, IPIavg = average IPI, IPIsd = standard deviation of the IPI, IPIvar = variance of the IPI, IPIshannon = Shannon entropy of the IPI, SNRr = adjusted rsquared for the regression on the signal SNR, SNRmin = minimum SNR, SNRmax = maximum SNR, SNRavg = average SNR, SNRsd = standard deviation of the SNR, SNRvar = variance of the SNR, slopeavg = average slope, slopemin = minimum slope, slopesd = standard deviation of the slope, duravg = average call duration, dursd = standard deviation of the call duration, smoothavg = average of the smoothness parameter, smoothvar = variance of the smoothness parameter.
