# Supplementary material for: Buzzfindr: Automating the detection of feeding buzzes in bat echolocation recordings
Source: PLoS One. 2024 Aug 20;19(8):e0306063. doi: 10.1371/journal.pone.0306063 (PMC11335113; doi:10.1371/journal.pone.0306063)
Supplement: S5 File — The accuracy in detecting feeding buzzes in test-data (i.e. proportion of accurately classified passes) was examined for three classification methods (LDA: Linear discriminant analysis; RF: Random forests; ANN: Artificial neural networks) at incremental detection threshold levels. (PDF) [file pone.0306063.s005.pdf]

**S5 File. Accuracy of three modelling methods in detecting feeding buzzes in test-data.** The accuracy in detecting feeding buzzes in test-data (i.e. proportion of accurately classified passes) was examined for three classification methods (LDA: Linear discriminant analysis; RF: Random forests; ANN: Artificial neural networks) at incremental detection threshold levels.

| Threshold | LDA | RF | ANN |
|---|---|---|---|
| 0.50 | 0.96 | 1.00 | 1.00 |
| 0.55 | 0.96 | 1.00 | 1.00 |
| 0.60 | 0.96 | 1.00 | 1.00 |
| 0.65 | 0.95 | 1.00 | 1.00 |
| 0.70 | 0.95 | 1.00 | 1.00 |
| 0.75 | 0.94 | 0.99 | 1.00 |
| 0.80 | 0.94 | 0.98 | 1.00 |
| 0.85 | 0.93 | 0.97 | 1.00 |
| 0.90 | 0.91 | 0.96 | 0.99 |
| 0.95 | 0.87 | 0.95 | 0.98 |
| 1.00 | 0.50 | 0.70 | 0.50 |
