# Supplementary material for: Buzzfindr: Automating the detection of feeding buzzes in bat echolocation recordings
Source: PLoS One. 2024 Aug 20;19(8):e0306063. doi: 10.1371/journal.pone.0306063 (PMC11335113; doi:10.1371/journal.pone.0306063)
Supplement: S6 File — Results from a logistic regression testing the effect of recorder type (SM2BAT+, SM4BAT, Bat Mini) and species frequency group (High-frequency, Low-frequency) on the accuracy of the classifier. (PDF) [file pone.0306063.s006.pdf]

S6 File. Effect of recorder type and species frequency group on classifier accuracy. Results from a logistic regression testing the effect of recorder type (SM2BAT+, SM4BAT, Bat Mini) and species frequency group (High-frequency, Low-frequency) on the accuracy of the classifier.

Model Parameter	Estimate	Standard Error	z-value	P-value
(Intercept)	2.51	0.33	7.63	<0.001
Recorder (SM2BAT+)	-0.14	0.45	-0.32	0.750
Recorder (SM4BAT)	0.53	0.49	1.09	0.274
Frequency Group (Low-frequency)	-0.13	0.45	-0.29	0.776
Recorder (SM2BAT+) : Frequency Group (Low-frequency)	0.36	0.62	0.58	0.564
Recorder (SM4BAT) : Frequency Group (Low-frequency)	0.18	0.71	0.26	0.799
